# The Psychological Impact of the COVID-19 Pandemic on Alcohol Abuse and Drunkorexia Behaviors in Young Adults

**DOI:** 10.3390/ijerph20043466

**Published:** 2023-02-16

**Authors:** Daniele Di Tata, Dora Bianchi, Sara Pompili, Fiorenzo Laghi

**Affiliations:** Department of Developmental and Social Psychology, Sapienza University of Rome, 00185 Rome, Italy

**Keywords:** drunkorexia, food and alcohol disturbance, alcohol abuse, COVID-19 pandemic, young adults

## Abstract

The COVID-19 outbreak negatively affected young adults’ psychological well-being, increasing their stress levels and symptoms of anxiety and depression, and potentially triggering health-risk behaviors. The present study was aimed at investigating the psychological impact of the COVID-19 pandemic on alcohol abuse and drunkorexia behaviors among young adults living in Italy. Participants were 370 emerging adults (63% women, 37% men; *M*_age_ = 21.00, *SD*_age_ = 2.96, range: 18–30) who were recruited through an online survey between November 2021 and March 2022. Participants completed measures of alcohol abuse, drunkorexia behaviors, negative life experiences, and post-traumatic symptoms related to the COVID-19 outbreak. The results showed that the emotional impact and negative life experiences associated with the pandemic predicted both alcohol abuse and drunkorexia behaviors, albeit in different ways. Specifically, the number of negative life experiences during the pandemic and the tendency to avoid COVID-19–related negative thoughts positively predicted alcohol abuse; and the presence of intrusive thoughts associated with the pandemic significantly predicted the frequency of drunkorexia behaviors. Implications for research and clinical practice are discussed.

## 1. Introduction

The COVID-19 pandemic and the emergency measures adopted to counteract it profoundly impacted lifestyles, with negative effects on psychological well-being [1]. In addition to generating health risks and concerns, the COVID-19 pandemic also led to feelings of instability and insecurity towards the future and imposed dramatic and sudden changes in social, academic, and professional realms. For example, the onset of the outbreak in 2020 quickly caused a global economic crisis, increasing unemployment rates worldwide and leaving millions of people suddenly furloughed or unemployed. Moreover, schools and universities closed for a long period, forcing the education system to transition to online learning. Additionally, the social restrictions adopted to combat the pandemic negatively impacted individuals’ ability to spend quality time with others, including friends and romantic partners. This situation may have been particularly stressful for young adults, who were already facing challenging and complex age-related tasks, such as achieving independence from the family, working towards career goals, and developing romantic relationships [2,3]. Thus, the forced isolation, home confinement, and other strict pandemic rules may have interfered with the developmental tasks of young adulthood, generating stress and insecurity in youths [4]. In line with this, research has shown that, although older adults were more at risk of contracting a severe form of the disease, emerging adults reported relatively increased concerns about the health of their loved ones [5] and greater vulnerability to pandemic-related stress [6].

Studies have found a higher prevalence of psychological problems, such as anxiety, depression, and mood disorders among emerging adults during the COVID-19 outbreak, compared with pre-pandemic levels [7,8,9,10]. These emotional issues may have had cascade effects on health risk behaviors, increasing the likelihood that young adults would develop alcohol-related problems and dysfunctional eating patterns, such as those associated with drunkorexia. The long-term psychological impact of the COVID-19 pandemic on drunkorexia behaviors has not yet been studied, despite recent evidence that maladaptive emotion regulation strategies significantly predicted drunkorexia behaviors during the lockdown [11]. Moreover, a trend of increased alcohol use during the pandemic [12,13] highlights the need to identify COVID-19–specific distress factors associated with alcohol abuse in order to better prevent and monitor these behaviors in the post-pandemic phase.

### 1.1. Alcohol Use and the COVID-19 Pandemic

Studies have produced mixed results with regard to changes in alcohol consumption during the COVID-19 pandemic [12]. Some studies have reported a decrease in alcohol use due to the social restrictions imposed by governments to prevent contagion [14,15,16], while others have shown that drinking behaviors increased in individuals who used alcohol to cope with pandemic-related distress [17,18]. Both of these patterns are aligned with the motivational model of alcohol use [19,20,21], which suggests that drinking behaviors are driven by different needs and serve several functions. For instance, individuals may drink to achieve a positive outcome (e.g., social acceptance, peer approval) or to avoid a negative one. According to this model, the COVID-19 health emergency affected individuals’ motivation to drink in different ways: on the one hand, social drinking was reduced by the limits imposed on social gatherings, while on the other hand, emotional distress associated with the pandemic may have increased emotional drinking, as a method of coping.

In addition, the interaction between different motivations to drink and the lifestyle changes imposed by the pandemic must be considered. During emerging adulthood, the need to be socially accepted and connected with others may facilitate alcohol consumption episodes, often during parties or small social gatherings. In this vein, the reduction of social activities due to the health emergency may have contributed to decreased alcohol use among youths [14]. Nevertheless, studies have shown that the COVID-19 pandemic negatively impacted young adults’ psychological well-being and mental health [6,7,8], and this may have led some youths to use substances (e.g., alcohol) to alleviate distress and negative feelings associated with the dramatic situation. For instance, Vera et al. [22] documented that young people with higher levels of depression were more resistant to decreasing their alcohol consumption during the pandemic relative to the general population.

Research has not yet examined the possible long-term effects of pandemic-related emotional symptoms on alcohol abuse patterns in young people, although drinking for coping was highly documented in research conducted during the COVID-19 outbreak [17,18,23,24]. The self-medication hypothesis (SMH) [25] proposes that individuals with difficulty self-regulating may use alcohol to relieve or alter their subjective state of distress. Specifically, the anesthetic effect of alcohol could supplant the inability of some individuals to recognize and regulate intolerable emotions. Moreover, several studies have provided support for the SMH regarding trauma-related drinking to cope (see [26] for a review). In particular, research has shown significant associations between post-traumatic symptoms and a coping motivation to drink [27]. Relative to the COVID-19 pandemic, studies have found an association between higher levels of psychological distress and increased alcohol consumption during the health emergency [28,29]. However, evidence is lacking on the long-term effect of COVID-19 trauma-related symptoms on alcohol abuse.

### 1.2. Drunkorexia Behaviors and the COVID-19 Pandemic

As Rodgers and colleagues [30] suggested, the COVID-19 pandemic also exacerbated problem eating behaviors, increasing risk conditions, and decreasing protective factors. During the pandemic, individuals experienced higher levels of emotional distress, anxiety, and depression symptoms [31], which are triggering factors for problematic eating behaviors [32]. Conversely, the protective effects of emotional and social support were reduced by the forced isolation. As a consequence, there was an increase in the incidence of eating disorders characterized by binge and restrictive behaviors (e.g., bulimia nervosa and anorexia, respectively) [33,34,35,36,37,38], and a worsening of symptoms in individuals who had already been experiencing these conditions [39,40].

Drunkorexia (also known as food and alcohol disturbance) refers to a pattern of restrictive and compensatory behaviors that are practiced when drinking is planned, in order to compensate for the calories consumed and/or to enhance the intoxicating effect of the alcohol [41,42,43]. These behaviors include self-imposed calorie restriction, fasting, purging, and excessive exercising [44]. Research has identified several commonalities between drunkorexia and eating disorders [45], suggesting the presence of similar dysfunctional patterns [46,47,48]. Drunkorexia behaviors have also been shown to be positively associated with affective variables, including emotion dysregulation, psychological distress, and post-traumatic symptoms [49,50,51,52,53]. Thus, drunkorexia can be considered an eating disorder combined with alcohol use, related to negative emotional experiences and maladaptive coping strategies.

Research has shown that drunkorexia is very common among young adults and is mostly driven by a motivation to drink alcohol in order to cope [54,55]. Despite recent evidence of the pandemic’s role in contributing to an upsurge in eating disorders, drinking problems, and other negative psychological conditions [7,12,16,30,40], little is known about the impact of pandemic-related stress on drunkorexia behaviors [11]. However, recent studies have documented an association between drunkorexia, psychological distress, and emotion regulation difficulties [56,57,58,59]. For instance, Qi and colleagues [59] observed higher mean scores of affective problems (e.g., anxiety, depression) in young adults engaging in drunkorexia behaviors. Thus, it is worth exploring the possible impact of the COVID-19 pandemic and the emotional consequences of the health emergency on young adults’ drunkorexia behaviors.

### 1.3. The Current Study

To fill this gap in the literature, the present study aimed to examine the psychological impact of the COVID-19 pandemic on alcohol abuse and drunkorexia behaviors in a sample of young adults living in Italy. Specifically, the effects of the number of COVID-19–related negative experiences in the prior year and their long-term emotional effects, as experienced during the prior week, were examined. The study aimed at investigating the predictive roles played by COVID-19-related experiences and two long-term emotional symptoms (i.e., intrusive thoughts about the pandemic, and avoidance of pandemic thoughts) on young adults’ alcohol abuse and drunkorexia behaviors, respectively.

Given the trend of increased alcohol consumption during the pandemic [12], positive associations were expected between alcohol abuse and both COVID-19–related negative experiences (H1) and the investigated long-term emotional symptoms (H2). In line with this, previous studies [60,61] have shown a significant positive relationship between alcohol use and COVID-19–specific distress factors, as well as increased alcohol consumption in individuals with psychological problems [62,63,64]. Moreover, based on evidence of a relationship of drunkorexia with psychological distress [51,59] and post-traumatic stress symptoms [52], positive associations were expected between drunkorexia behaviors and both COVID-19–related negative life experiences (H3) and the investigated long-term emotional symptoms (H4).

## 2. Materials and Methods

### 2.1. Participants and Procedure

In total, 370 emerging adults living in Italy participated in the study. In accordance with Arnett’s definition of emerging adulthood [2,3], participants included men and women aged 18–30 years. The inclusion criteria were: (a) aged 18–30 years, (b) currently living in Italy, and (c) at least occasionally drinking alcohol. Data were gathered from November 2021 to March 2022, and the recruitment was conducted online using a snowball sampling method. A link to the anonymous survey was disseminated via the university website, and each participant was asked to share the link with their acquaintances and friends. The first page of the survey explained the research procedures and guaranteed complete anonymity and voluntariness of participation. Participants indicated their informed consent before proceeding with the survey. The remainder of the online questionnaire took approximately 20 min to complete. Initially, 414 youths provided informed consent and completed the entire questionnaire. Of these, 40 were removed because they did not drink alcohol (i.e., exclusion criterion). Moreover, 4 participants were excluded for not correctly completing the survey. Therefore, only 370 young adults met all the criteria for inclusion in the research, representing a response rate of 89.4%. The study and its procedure were reviewed and approved by the Ethics Committee of the Department of Developmental and Social Psychology, Sapienza University of Rome.

Power analyses were conducted using G*Power software, version 3.1. Considering the conventional 80% power and 0.05 alpha significance level [65], the a priori power analysis indicated a required sample size of 311 to detect small effect sizes (Cohen’s d = 0.20).

### 2.2. Measures

#### 2.2.1. Individual Information

Participants reported their biological sex (0 = man; 1 = woman), age, country of origin, area of residence, and education level.

#### 2.2.2. Alcohol Abuse

The Alcohol Use Disorders Identification Test (AUDIT) [66,67] was used to assess participants’ alcohol abuse. The 10-item scale was designed by the World Health Organization to evaluate hazardous and harmful drinking. Items investigate the amount and frequency of alcohol intake, the presence of alcohol dependence symptoms, and the presence of alcohol-related problems, with answers rated on a 5-point scale ranging from 0 (never) to 4 (frequently or daily). The present study used the AUDIT mean total score. Male participants with a score ≥ 8 and female participants with a score ≥ 6 were considered high-risk drinkers in accordance with the guidelines set by previous studies [68,69]. The AUDIT has been shown to have good psychometric properties in the Italian context [29,69,70]. The present study confirmed the high reliability of the scale (Cronbach’s alpha = 0.80).

#### 2.2.3. Drunkorexia

The frequency of drunkorexia was evaluated using the Drunkorexia Behaviors subscale (12 items; sample item: “*On a day I planned to drink, I controlled my eating by avoiding fatty foods*”) of the Drunkorexia Motives and Behaviors Scale (DMBS) [71]. On this subscale, questions investigate the frequency with which compensatory behaviors are engaged around alcohol consumption episodes, with answers rated on a Likert-type scale ranging from 1 (*never*) to 5 (*always*). The DMBS has been shown to have good psychometric properties in the Italian context [11,12,13,14,15,16,17,18,19,20,21,22,23,24,25,26,27,28,29,30,31,32,33,34,35,36,37,38,39,40,41,42,43,44,45,46,47,48,49,50,51,52,53]. In the present study, the instrument showed high reliability (Cronbach’s alpha = 0.97). 

#### 2.2.4. COVID-19-Related Negative Experiences

A 19-item checklist was used to assess the number of stressors experienced by participants during the prior year due to the COVID-19 pandemic (i.e., “*Please answer the following questions about your experiences with COVID-19 over the past year*”). The instrument was inspired by the 16-COVID Stressors Checklist [72], which was modified to accommodate the Italian context and to meet the needs of the present study. Three additional items were added from the SARS-related Stressors Checklist [73]. Items 1–12 assessed participants’ experiences and whether their relatives, friends, and/or acquaintances had been infected (or were suspected of having been infected) by the virus (e.g., “*During the last year… did you contract COVID-19 and test positive for a swab?*”). Questions 13–16 assessed the degree to which participants had experienced distress due to information they had received about COVID-19 (e.g., “*Have you had a hard time understanding the authenticity of online information regarding the COVID-19 pandemic?*”), and questions 17–19 assessed the impact of the pandemic on participants’ daily life (e.g., “*Have you had conflicts and quarrels with your family due to the pandemic?*”). Each item had a dichotomous response option to indicate whether the COVID-19–related negative event had been experienced (0 = no; 1 = yes). The total score indicated the number of stressors participants had experienced in the prior year due to the pandemic.

#### 2.2.5. Psychological Impact of the COVID-19 Pandemic

The Impact of Event Scale with Modifications for COVID-19 (IES-COVID-19) [74] was administered to assess the long-term psychological impact of the pandemic on participants over the prior seven days. The original version of the instrument comprises 15 items, measuring two dimensions: Avoidance, which investigates the tendency to avoid COVID-19–related negative thoughts (8 items; sample item: “*Over the last seven days … I stayed away from things that made me think about the COVID-19 pandemic*”); and Intrusion, which assesses the presence of negative intrusive thoughts regarding the COVID-19 pandemic (7 items; sample item: “*Over the last seven days … I thought about the COVID-19 pandemic when I didn’t mean to*”). Answers are rated on a 4-point scale ranging from 1 (never) to 4 (often).

In the absence of an Italian version of the instrument, a blinded forward-backward translation procedure was conducted to adapt the original Dutch version to the Italian cultural context (guidelines by [75]). The few discrepancies that emerged were discussed by psychology researchers in a focus group. Thereafter, the Italian version of the IES-COVID-19 was included in the present study. A confirmatory factor analysis (CFA) was run on the data using the Jamovi software (version 2.3.16) to verify the adequacy of the two-factor model for Italian participants. Goodness-of-fit was estimated by the relative chi-square test statistic (*χ*^2^/df, values expected to range between 1–3 for good model fit) [76]; by the CFI, NFI, and NNFI indices (≥0.90 for acceptable fit) [77]; and by the RMSEA and SRMR indices (≤0.08 for acceptable fit) [78]. One item (i.e., item 15) was removed from the Italian version to improve the reliability of the two dimensions, and the final 14-item model obtained acceptable fit indices, confirming the presence of two correlated factors: *χ*^2^ (71) = 209.057, *p* < 0.001; *χ*^2^/df = 2.94; CFI = 0.94; NFI = 0.91; NNFI = 0.92; RMSEA = 0.07; 90% CI [0.06, 0.08]; SRMR = 0.05. The two factors were positively correlated (Pearson’s *r* = 0.51, *p* < 0.001) and showed good reliability in the Italian sample (Cronbach’s alphas of 0.84 for Avoidance and 0.86 for Intrusion).

### 2.3. Data Analysis

Data were analyzed using the statistical package SPSS, version 27. To preliminarily investigate the effects of gender and age differences on alcohol abuse and drunkorexia behaviors, two univariate analyses of covariance (ANCOVA) were performed, including gender as a between-subjects factor and age as a covariate. In the first ANCOVA, a continuous score of problematic alcohol use was entered as a dependent variable. In the second ANCOVA, drunkorexia was included as a dependent variable.

Bivariate Pearson’s correlations were computed among the study variables, with both drunkorexia and alcohol abuse included as continuous scores. Subsequently, to verify the first and second research hypotheses, a hierarchical regression analysis was performed on the continuous score of alcohol abuse. Gender and age were entered as covariates in step 1, while COVID-19–related negative experiences, Avoidance, and Intrusion were added to the regression equation in step 2. Finally, to investigate the third and fourth research hypotheses, a negative binomial regression analysis was conducted to test the effects of COVID-19–related experiences and emotional symptoms on the frequency of drunkorexia behaviors. Gender, age, and alcohol abuse were included in the model to control for their effects, and COVID-19–related negative experiences, Intrusion, and Avoidance were added to test the hypotheses.

## 3. Results

Participants were 370 young adults (*M*_age_ = 21.00, *SD*_age_ = 2.96, age range: 18–30; 63% women, 37% men) living in Italy. In terms of geography, 67% of participants resided in central Italy, 26% in southern Italy, and 7% in northern Italy. Most participants (97%) had been born in Italy, while 3% had an immigrant background. Regarding education level, 2% had finished middle school, 82% had graduated from high school, and the remaining 16% held a bachelor’s degree; moreover, 98% currently were university students.

Regarding the alcohol abuse groups, 149 participants (40%) were classified as “high-risk drinkers” and 221 as “low-risk drinkers,” based on the gender-adjusted cut-off scores for the AUDIT questionnaire. As regards drunkorexia, 139 participants (38%) reported enacting compensatory behaviors around episodes of alcohol consumption.

The results of the first ANCOVA on alcohol abuse indicated significant univariate effects of gender, *F*(1) = 9.45, *p* < 0.01, *η*^2^ = 03, with men reporting a higher mean score than women, and age, *F*(1) = 7.44, *p* < 0.01, *η*^2^ = 02.

With respect to drunkorexia behaviors, the results of the second ANCOVA showed a significant univariate effect of age, *F*(1) = 6.88, *p* < 0.01, *η*^2^ = 02. Net of this effect, a significant difference between men and women was found, *F*(1) = 6.11, *p* < 0.05, *η*^2^ = 02. Specifically, women reported higher scores on drunkorexia relative to men (see Table 1).

Table 2 summarizes the descriptive statistics and bivariate Pearson’s correlations. The analysis showed a significant and positive correlation between alcohol abuse and drunkorexia behaviors, with both behaviors positively and significantly associated with the number of COVID–related negative experiences during the health emergency, the presence of negative intrusive thoughts about the pandemic, and the tendency to avoid such negative thoughts.

Regarding the hierarchical regression analysis on the alcohol abuse continuous score, the results in step 1 explained 3% of the variance in alcohol abuse, detecting significant negative effects of gender (with men reporting higher scores than women) and age (with younger people reporting higher scores). Step 2 contributed a significant 6% to the explained variance. Gender and age were still significant covariates, and controlling for their effects, COVID-19–related negative life experiences and Avoidance significantly and positively predicted alcohol abuse. Conversely, Intrusion was not significant. The final model explained 9% of the variance in alcohol abuse (see Table 3 for statistics).

The negative binomial regression model explained a significant 17% of the variance, detecting a significant effect of gender (with women reporting higher scores) and age (with younger participants reporting higher rates of drunkorexia). Moreover, alcohol abuse showed a significant and positive effect on drunkorexia behaviors. Net of these effects, the presence of intrusive negative thoughts about the pandemic emerged as a significant predictor of drunkorexia behaviors, whilst the effects of COVID-19 experiences and Avoidance were null. Table 4 reports the full model statistics.

## 4. Discussion

The present study investigated the psychological impact of the COVID-19 pandemic on alcohol abuse and drunkorexia behaviors in young adults. Specifically, the number of COVID-19–related negative experiences, the presence of negative intrusive thoughts regarding the pandemic, and the tendency to avoid these negative thoughts were explored to understand their roles in predicting these two health-risk behaviors. Recent studies have observed an increase in alcohol consumption and eating disorders during the COVID-19 pandemic [12,35]. Thus, it is reasonable to expect a long-term emotional impact of the pandemic on alcohol abuse and drunkorexia behaviors. To the best of our knowledge, the present study was the first to investigate the relationship between drunkorexia and COVID-19–related stressors and emotional symptoms.

The results of the preliminary analyses showed that women reported more frequent drunkorexia behaviors compared to men. This finding contradicts the results of previous international studies finding no gender differences in drunkorexic tendencies [79]. Conversely, men showed higher levels of alcohol abuse than women, in line with recent research on young Italians during the COVID-19 pandemic [80]. Moreover, the preliminary analyses found that younger participants reported higher scores for both drunkorexia behaviors and alcohol abuse.

The first regression analysis found a significant effect of age (with younger participants reporting higher rates) and gender (with men reporting higher rates than women) on alcohol abuse. These findings confirm the results of previous studies that have found men to be significantly more likely to use alcohol than women during the pandemic [14,81,82]. Regarding age, previous research has produced mixed results, with most studies indicating higher alcohol consumption in older people during the COVID-19 outbreak [12]. However, the relaxation of containment measures in the last year recreated social drinking opportunities for younger people, who consequently reported a greater tendency to engage in problematic alcohol use. Controlling for gender and age effects, alcohol abuse was significantly and positively predicted by COVID-19–related negative life experiences and the avoidance of thinking about these events. These findings suggest that pandemic-related stress may have contributed to increased alcohol consumption aimed at coping with negative emotions [18,83], in line with the SMH [25]. Specifically, both the higher number of negative stressors caused by the pandemic and the tendency to avoid COVID-19–related negative thoughts predicted higher alcohol abuse, suggesting that alcohol consumption served as a maladaptive emotion regulation strategy in response to negative experiences. However, the percentage of variance explained by the model (9%) suggests that the results should be interpreted with caution. Other predictors not considered by the study (e.g., lack of social support and mental health problems) may also contribute to explaining the risk of alcohol abuse related to the pandemic. Thus, future studies should investigate the relationship between alcohol use and COVID-19–related psychological distress more deeply by including more control variables (e.g., mental health problems, perceived social support, and living conditions during the pandemic).

Regarding the second regression analysis on drunkorexia behaviors, a significant association with gender was found, with women reporting a greater likelihood of engaging in compensatory behaviors around an episode of alcohol consumption relative to men. This finding aligns with the results of previous research [84]. A significant negative effect of age on drunkorexia behaviors was also found, with younger participants reporting a significantly greater likelihood of restricting calories when deciding to drink alcohol, suggesting that drunkorexia risk may decrease with age. Indeed, previous research has shown that risky behaviors are more frequent during adolescence and emerging adulthood and decrease with the transition into adulthood [85].

In line with previous studies [45,46,47,48,49,50,51,52,53,54,55,56,57,58,59,60,61,62,63,64,65,66,67,68,69,70,71,72,73,74,75,76,77,78,79,80,81,82,83,84,85,86], the present study found that alcohol abuse positively predicted drunkorexia, whereby participants with greater alcohol-related problems exhibited more frequent dysfunctional compensatory behaviors with respect to COVID-19–related variables, the long-term emotional impact of the pandemic also affected drunkorexia behaviors, albeit in a very different way, confirming the uniqueness of this behavior. Specifically, only the frequency of negative intrusive thoughts about the pandemic positively predicted drunkorexia behaviors, while avoidance of these thoughts and the number of COVID-19 negative experiences were not significant. Thus, drunkorexia was predicted by ruminative thinking about the pandemic, regardless of the actual negative events that were experienced. Previous studies have shown that ruminative thoughts predict different eating disorders in the short and long term (review by [87]). The present findings uncovered a similar pattern in drunkorexic tendencies. This result, together with the very different findings of the first regression on alcohol abuse, provides new evidence for the higher affinity of drunkorexia with other eating disorders rather than alcohol-related problems. Moreover, the present findings corroborate previous evidence showing that psychological distress is a risk factor for engaging in compensatory behaviors around episodes of alcohol consumption [46,88].

## 5. Conclusions

The present study represented one of the first attempts to explore the long-term emotional impact of the COVID-19 pandemic on alcohol abuse and drunkorexia behaviors. However, it also suffered from some limitations. First, data were obtained through self-report instruments, which may have been affected by social desirability bias. Consequently, some undesirable behaviors (e.g., alcohol misuse) might have been underreported. Second, the data were collected in Italy, and thus, the findings may not be generalizable to populations of countries with different experiences of the pandemic. Moreover, participants were not representative of the target population but constituted a convenience sample recruited through the dissemination of an anonymous online questionnaire. Third, due to the cross-sectional research design, only correlational associations between study variables, and not causal relationships, could be drawn. Future studies should attempt to confirm the present results with representative samples from different countries. In addition, future research should continue to monitor the psychological impact of the pandemic on health-risk behaviors among young people by implementing mixed research methodologies.

Despite these study limitations, the present results provide useful information for the implementation of prevention programs to monitor alcohol abuse and drunkorexia behaviors in the post-pandemic phase, helping to identify at-risk individuals. It is recommended that public health interventions focus on reducing psychological distress and supporting individuals who suffered from the pandemic to prevent further problematic behavioral consequences.

## Figures and Tables

**Table 1:** Descriptive statistics on alcohol abuse and drunkorexia.

		Men	Women	Total
	Range	*M (SD)*	*M (SD)*	*M (SD)*
Alcohol Abuse	1–25	6.93 (4.61)	5.81 (4.25)	6.23 (4.42)
Drunkorexia	1–5	1.20 (0.43)	1.47 (0.89)	1.37 (0.76)

**Table 2:** Descriptive statistics and bivariate Pearson’s correlations on study variables.

	1	2	3	4	5	6	7	Range	*M (SD)*
1. Gender (0 = Male, 1 = Female)	1							-	-
2. Age	−0.29 **	1						18–30	21 (2.96)
3. COVID-19 related negative experiences	0.06	−0.26 **	1					0–19	13.29 (3.33)
4. Avoidance	0.19 **	−0.06	0.21 **	1				1–4	1.84 (0.71)
5. Intrusion	0.14 **	−0.02	0.35 **	0.48 **	1			1–3.75	1.81 (0.64)
6. Alcohol abuse	−0.12 *	−0.10	0.17 **	0.17 **	0.13 *	1		1–25	6.23 (4.42)
7. Drunkorexia	0.17 **	−0.18 **	0.13 *	0.13 **	0.16 **	0.25 **	1	1–5	1.37 (0.76)

Notes: ** *p* < 0.01; * *p* < 0.05. Correlations were computed on the continuous scores for drunkorexia and alcohol abuse.

**Table 3:** Hierarchical regression model predicting alcohol abuse.

	Alcohol Abuse			
	Step 1		Step 2	
Predictors	*R^2^*	*beta*	Δ*R^2^*	*beta*
	0.03 *		0.06 **	
Gender (0 = Male; 1 =Female)		−0.16 **		−0.20 **
Age		−0.15 **		−0.12 *
COVID-19 related negative experiences				0.11 *
Avoidance				0.16 **
Intrusion				0.03
Total *R*^2^	0.09 **			

Notes: ** *p* ≤ 0.01; * *p* ≤ 0.05. Standardized regression coefficients are reported.

**Table 4:** Negative binomial regression model on variables predicting drunkorexia.

	Drunkorexia	
Predictors	*B (SE)*	*OR [95% CI]*
Male ^a^ vs. female	0.16 (0.05) **	1.18 [1.07–1.30]
Age	−0.02 (0.01) *	0.98 [0.96–1.00]
Alcohol Abuse	0.03 (0.01) **	1.03 [1.02–1.04]
COVID-19 related negative experiences	0.00 (0.01)	1.00 [0.99–1.02]
Avoidance	−0.00 (0.04)	1.00 [0.93–1.07]
Intrusion	0.08 (0.04) *	1.09 [1.00–1.18]
McFadden *R^2^*	0.17 *	

Notes: *SE*: standard error; *OR*: odds ratio; *CI*: confidence interval; ^a^ Reference category for independent variables; * *p* < 0.05; ** *p* < 0.01.

## Data Availability

Data are available under request to the first author.
